# Higher empathy is associated with stronger social bonding when moving together with music

**DOI:** 10.1177/03057356211050681

**Published:** 2021-11-30

**Authors:** Jan Stupacher, Jannie Mikkelsen, Peter Vuust

**Affiliations:** 1 Center for Music in the Brain, Department of Clinical Medicine, Aarhus University & The Royal Academy of Music, Aarhus/Aalborg, Aarhus C, Denmark; 2 School of Business and Social Sciences, Aarhus University, Aarhus C, Denmark

**Keywords:** empathy, social bonding, music, synchronization, entrainment, joint action, affiliation

## Abstract

Empathy—understanding and sharing the feelings and experiences of others—is one of our most important social capacities. Music is a social stimulus in that it involves communication of mental states, imitation of behavior, and synchronization of movements. As empathy and music are so closely linked, we investigated whether higher empathy is associated with stronger social bonding in interpersonal interactions that feature music. In two studies, participants watched videos in which we manipulated interpersonal synchrony between the movements of a virtual self and a virtual other person during walking with instrumental music or a metronome. In both studies, temporally aligned movements increased social bonding with the virtual other and higher empathy was associated with increased social bonding in movement interactions that featured music. Additionally, in Study 1, participants with lower empathy felt more connected when interacting with a metronome compared to music. In Study 2, higher trait empathy was associated with strong increases of social bonding when interacting with a temporally aligned virtual other, but only weak increases of social bonding with a temporally misaligned virtual other. These findings suggest that empathy plays a multifaceted role in how we enjoy, interpret, and use music in social situations.

Empathy is a multidimensional concept that describes the understanding and sharing of viewpoints and emotions of others. Representing some of the most central aspects of social functioning, empathy results from the interplay between trait capacities and state influences (Cuff et al., 2016) comprising an individual’s cognitive, perspective-taking capabilities, and emotional reactivity (Davis, 1980). High empathy is positively associated with prosocial behavior (Eisenberg & Miller, 1987; Paciello et al., 2013) and negatively associated with social prejudice (Batson et al., 1997; Miklikowska, 2018).

Music is a social phenomenon in that we make, listen to, and dance to music together. In musical activities, we express attitudes, elicit emotions, imitate behavior, and synchronize movements, creating a feeling of togetherness (Cross et al., 2012). The social nature of musical stimuli explains why empathy is important for understanding emotions in music (Eerola et al., 2016; Egermann & McAdams, 2013; Wöllner, 2012) and why moving synchronously with music can increase prosocial behavior, interpersonal closeness, and cooperation (Cirelli et al., 2014; Kirschner & Tomasello, 2010; Stupacher, Maes, et al., 2017; Stupacher, Wood, & Witte, 2017; Wiltermuth & Heath, 2009).

Previous research suggests that music listening and musical interactions can promote empathy (Clarke et al., 2015; Greenberg et al., 2015; Rabinowitch et al., 2013). Vice versa, more empathic individuals may be more sensitive to social information in musical contexts (Keller et al., 2014). Participant pairs with higher empathic perspective-taking scores were more synchronized in a joint music-making task than participant pairs with low empathic perspective-taking scores, suggesting that sensorimotor mechanisms are crucial for simulating and anticipating the actions of another person in social interactions that feature music (Novembre et al., 2019). However, findings of Carlson and colleagues (2019) suggest that the relationship between empathy and interpersonal movement synchronization in musical contexts might be less stable than one would assume. In contrast to Novembre and colleagues’ (2019) findings and against Carlson and colleagues’ hypotheses, dancing pairs in which both partners had high trait empathy were perceived as moving less similar and less interactive than dancing pairs in which one partner had high and the other low trait empathy. These divergent findings reveal that it remains a debated question how empathy affects social bonding and well-being when moving to music in pairs or groups.

We investigated whether individuals with high empathy experience stronger interpersonal closeness in movement interactions that feature music compared to individuals with low empathy. We tested this hypothesis in two studies that used a social entrainment video paradigm (Stupacher et al., 2020; Stupacher, Maes, et al., 2017) in which participants with individual differences in empathic responsiveness rated the interpersonal closeness toward a virtual partner moving temporally aligned or misaligned with the beat of music or a metronome. In Study 1 (*N* = 146), the musical stimulus consisted of one excerpt from a jazz trio piece (“Elevation of Love” by Esbjörn Svensson Trio). Study 2 (*N* = 162) included three musical excerpts from the genres pop, funk, and jazz to test the generalizability of the results.

## Study 1

### Method

#### Participants

We analyzed data of 146 participants (mean age = 27.8 years, *SD* = 8.6; 88 female, 57 male, 1 other) based on sample sizes of previous experiments using similar paradigms (Stupacher et al., 2020; Stupacher, Maes, et al., 2017). Eighty-eight participants were Danish; the other 58 participants had 30 different nationalities. One additional participant was excluded because they gave the lowest rating on every item of the empathy questionnaire. Informed consent was provided and the study was approved by the IRB at the Danish Neuroscience Centre.

#### Stimuli and design

**Figure 1.**
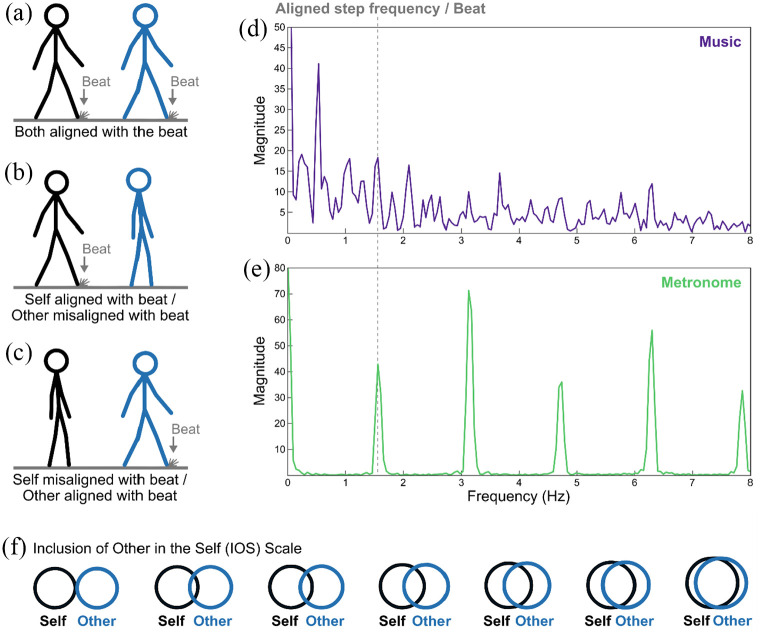
(a to c) The Three Different Movement Alignment Levels of the Social Entrainment Video Paradigm in Study 1. Participants Imagined That They Are the Black Figure and That the Blue Figure Represents an Unknown Other Person. (d and e) Frequency Spectra of the Note-Onset Interval Series of the Music and Metronome Stimuli Detected by *mironsets* in the MIR Toolbox (Lartillot & Toiviainen, 2007) for MATLAB (MathWorks, Natick, MA). The Dashed Gray Line Represents the Beat of the Audio Stimuli and the Temporally Aligned Step Frequency of the Visual Stimuli, Which Was 94 bpm/638 ms. The Temporally Misaligned Step Frequency Was 80 bpm/750 ms. (f) Adapted Inclusion of Other in the Self Scale (Aron et al., 1992).

Participants rated six videos with a length of 17 s each (see Supplemental material online). These videos showed the following two walking stick figures: a black one and a blue one. The black figure represented the participants—hereafter, referred to as virtual self—and the blue figure represented another person—hereafter, referred to as virtual other. The manipulated independent variables were *movement* and *music*. The variable *movement* had the following three levels: (1) both figures aligned with the beat, (2) self aligned with the beat and other misaligned, and (3) self misaligned with the beat and other aligned (Figure 1(a) to (c)). The variable *music* had the following two levels: (1) music and (2) metronome (Figure 1(d) and (e); Supplemental material online). The musical piece was an excerpt of “Elevation of Love” by Esbjörn Svensson Trio in 6/8 m with a tempo of 94 beats per minute (bpm)/638 ms when counting binary subdivisions with subdivisions at 188 bpm (Figure 1(d)). The metronome consisted of a snare drum with an inter onset interval of 94 bpm and a hi-hat with a tempo of 188 bpm (Figure 1(e)). Temporally aligned figures had a step frequency of 94 bpm and the foot hit the ground on the beat of the music or the metronome. The moment of the foot hitting the ground was additionally marked by a “dust cloud” on the ground (see Figure 1). Temporally misaligned figures had a step frequency of 80 bpm/750 ms with a 125 ms delayed first step. This manipulation of frequency and phase ensured that the misaligned movement combinations resulted in constantly changing phase relationships between the steps of the two figures.

#### Procedure and ratings

Participants watched each of the six videos in randomized orders and imagined that the black figure represented themselves and that the blue figure represented an unknown person (Stupacher et al., 2020; Stupacher, Maes, et al., 2017). After each video, they rated social bonding with the virtual other and their well-being as the virtual self.

Social bonding was operationalized as the dependent variable *Inclusion of Other in the Self* (IOS; Aron et al., 1992; Figure 1(f)). Participants chose a position on a 100-point visual analogue scale that best described the relationship between their virtual self and the virtual other person.

*Well-being* was measured by the question “How did you feel in this situation as the black figure?” that participants answered on a 100-point visual analogue scale. We included this dependent variable because Stupacher, Maes, and colleagues (2017) found an effect of movement synchrony and music on well-being in a similar paradigm. How well-being is affected by trait empathy in these types of virtual social interactions remains an open question. Additionally, well-being addresses a more introversive aspect of social interactions than the IOS.

After rating all six videos, participants rated the *familiarity with the music*, the *enjoyment of the music*, and the *perceived beat clarity of the music*. At the end of the survey, participants filled out the Brief Form of the Interpersonal Reactivity Index (B-IRI; Ingoglia et al., 2016). *Empathy* was operationalized as the overall score of the B-IRI including the subscales fantasy, perspective taking, empathic concern, and personal distress. Data were collected online on soscisurvey.de (SoSci Survey GmbH, Munich, Germany).

#### Data analysis

Using the lmer function from R’s (R Core Team, 2018) lme4 package (Bates et al., 2015), we modeled the dependent variables IOS and *well-being* in linear mixed effects models (Table 1). All models allowed for a random intercept per participant to control for participant level variability of *IOS* and *well-being* ratings. The full models included the following fixed effects: *Movement* (both aligned with the beat vs. self aligned/other misaligned vs. self misaligned/other aligned), *music* (music vs. metronome), and *empathy*.

**Table 1.** Mixed Effects Models for the Dependent Variables *Inclusion of Other in the Self* (IOS) and *Well-Being* Investigating the Effects of the Independent Variables *Movement*, *Music*, and *Empathy* in Study 1.

Model	AIC	BIC	Marg. *R*^2^	Cond. *R*^2^	Model fit improvement	
					χ^2^(*df*)	*p*
Dependent variable: IOS						
M0: IOS null model	8,345	8,359		.234		
M1: IOS ~ movement (vs. M0)	7,845	7,869	.318	.615	503.52 (2)	<.001
M2: IOS ~ movement + music (vs. M1)	7,843	7,872	.320	.617	4.27 (1)	.039
M3: IOS ~ movement × music (vs. M2)	7,846	7,884	.320	.617	0.90 (2)	.637
M4: IOS ~ movement + music + empathy (vs. M2)	7,845	7,878	.319	.618	0.02 (1)	.881
**M5: IOS ~ movement** **+** **music** × **empathy (vs. M2)**	**7,837**	**7,875**	**.323**	**.623**	**10.33 (2)**	**.006**
M6: IOS ~ movement × music × empathy (vs. M5)	7,847	7,914	.323	.622	1.25 (6)	.974
Dependent variable: Well-being						
M0: Well-being null model	8,175	8,189		.205		
M1: Well-being ~ movement (vs. M0)	8,001	8,025	.143	.376	177.40 (2)	<.001
M2: Well-being ~ movement + music (vs. M1)	7,967	7,996	.168	.405	35.97 (1)	<.001
M3: Well-being ~ movement × music (vs. M2)	7,968	8,007	.170	.407	3.05 (2)	.217
M4: Well-being ~ movement + music + empathy (vs. M2)	7,969	8,003	.167	.407	0.03 (1)	.863
**M5: Well-being ~ movement** **+** **music × empathy (vs. M2)**	**7,957**	**7,996**	**.177**	**.417**	**14.02 (2)**	**<.001**
M6: Well-being ~ movement × music × empathy (vs. M5)	7,966	8,033	.178	.418	3.70 (6)	.718

AIC: Akaike information criterion; BIC: Bayesian information criterion; IOS: inclusion of other in the self.

All models included a random effect for participants. The Akaike information criterion (AIC), Bayesian information criterion (BIC), variance explained by fixed effects (marginal *R*^2^), and variance explained by fixed and random effects (conditional *R*^2^) are provided. χ^2^ and *p* values refer to model comparisons against models indicated in parentheses using likelihood ratio tests. For both dependent variables, model M5 describes the data best (marked in bold).

### Results

**Figure 2.**
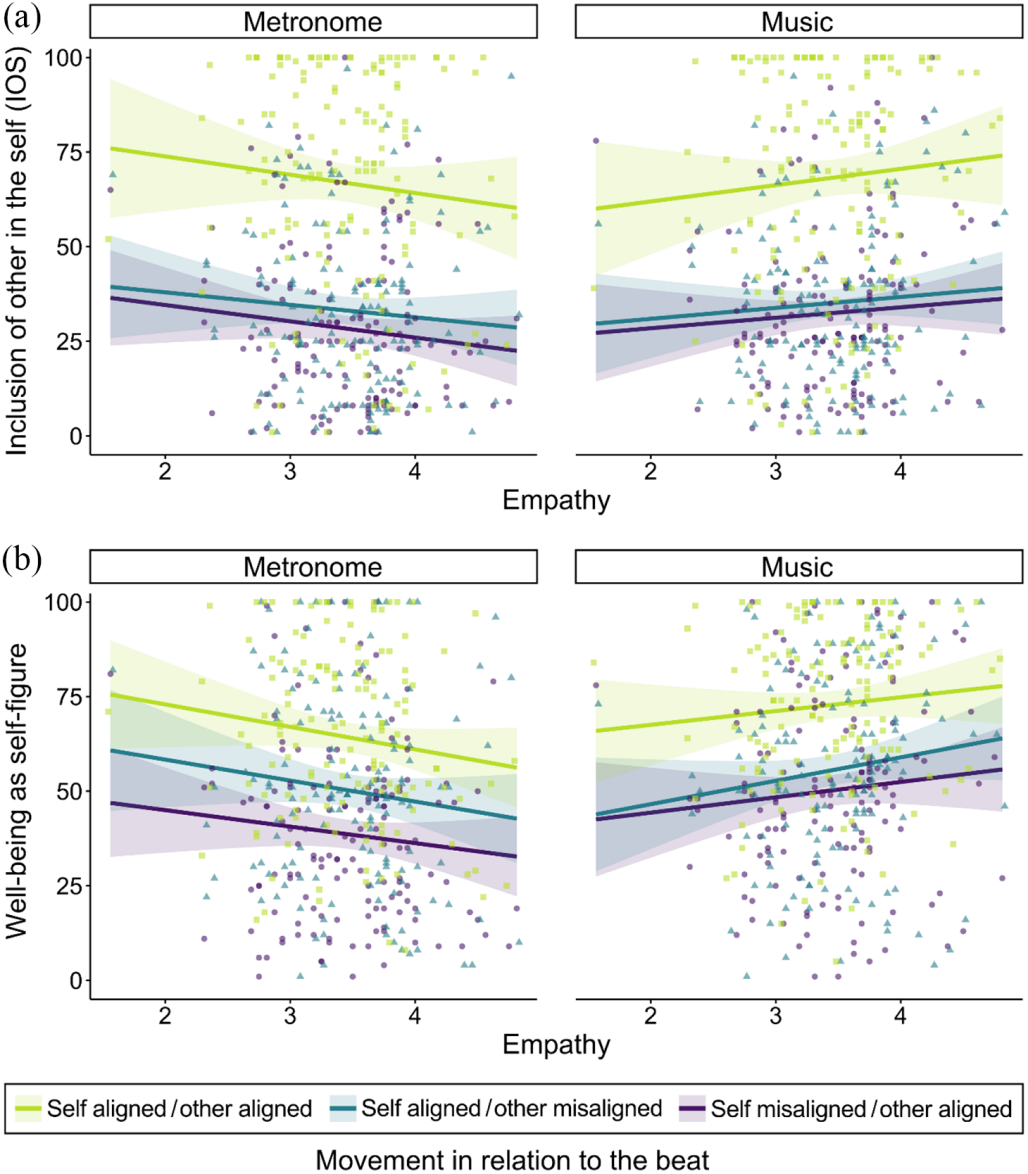
Individual Data Points and Linear Predictions of Interpersonal Closeness as Measured by *IOS* (Aron et al., 1992) and Well-Being in Relation to Participants’ Trait Empathy in Study 1. Shaded Areas Represent 95% Confidence Intervals. (a) *IOS* for All Three *Movement* Conditions in Interactions With Metronome and Music. (b) *Well-Being* for All Three *Movement* Conditions in Interactions With Metronome and Music.

Participants with higher empathy experienced higher interpersonal closeness in virtual movement interactions that featured music, whereas participants with lower empathy experienced higher interpersonal closeness in virtual movement interactions that featured a metronome, *t*(726) = 3.21, *p* = .006 (Table 2). Additionally, interpersonal closeness to the virtual other was higher for temporally aligned movements compared to misaligned movements (pairwise comparisons in Table 2). These results are reflected in the comparisons of linear mixed effects models for the dependent variable *IOS*: The best fitting model indicated an interaction between *music* and *empathy* and a main effect of *movement* (Figure 2(a), Table 1).

**Table 2.** Pairwise Comparisons of Individual Factor Levels in the Best Fitting Linear Mixed Effects Models in Study 1.

Model	Pairwise comparison	Estimate	*SE*	*df*	*t*	*p*	95% CI
IOS ~ movement + music × empathy	Self misaligned/other aligned—self aligned/other misaligned	−3.74	1.15	726	−2.48	.053	[−7.5, 0.03]
	Self misaligned/other aligned—self aligned/other aligned	−37.13	1.15	726	−24.63	<.001	[−40.9, −33.4]
	Self aligned/other misaligned—self aligned/other aligned	−33.39	1.15	726	−22.15	<.001	[−37.2, −29.6]
	Empathy|metronome—empathy|music (slope)	−7.47	2.33	726	−3.21	.006	[−13.3, −1.6]
Well-being ~ movement + music × empathy	Self misaligned/other aligned—self aligned/other misaligned	−8.67	1.68	726	−5.15	<.001	[−12.9, −4.5]
	Self misaligned/other aligned—self aligned/other aligned	−24.30	1.68	726	−14.43	<.001	[−28.5, −20.1]
	Self aligned/other misaligned—self aligned/other aligned	−15.64	1.68	726	−9.28	<.001	[−19.9, −11.4]
	Empathy|metronome—empathy|music (slope)	−9.74	2.60	726	−3.75	<.001	[−16.2, −3.2]

CI: confidence interval; IOS: inclusion of other in the self; *SE*: standard error.

Comparisons were computed using the emmeans package in *R* (Lenth et al., 2020). *p*-values and confidence intervals are Bonferroni-corrected for four comparisons.

Similarly, for the dependent variable *well-being*, the best fitting model indicated a main effect of *movement* and an interaction between *music* and *empathy* (Figure 2(b), Table 1). The significant interaction suggests that participants with higher empathy felt better when moving together with music, whereas participants with lower empathy felt better when moving together with a metronome, *t*(726) = 3.75, *p* < .001 (Table 2). The significant main effect of *movement* indicates that *well-being* was higher for temporally aligned movements compared to both misaligned movement conditions and higher for self aligned/other misaligned compared to self misaligned/other aligned (pairwise comparisons in Table 2).

**Figure 3.**
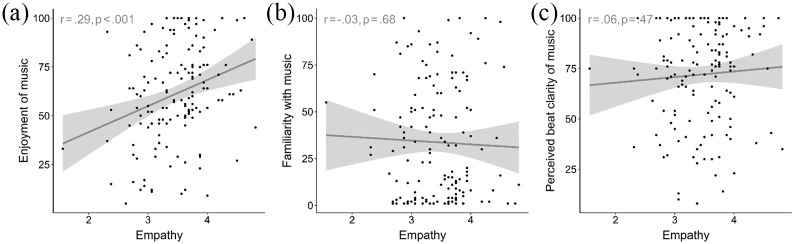
Individual Data Points and Linear Predictions of (a) Enjoyment of the Musical Stimulus, (b) Familiarity With the Musical Stimulus, and (c) Perceived Beat Clarity in Relation to Participants’ Trait Empathy in Study 1. Shaded Areas Represent 95% Confidence Intervals. Pearson’s Correlation Coefficients (*df* = 144) Are Provided in the Top Left Corners.

*Empathy* was positively correlated with overall *enjoyment of the music*, *r*(144) = .29, *p* < .001 (Figure 3(a)). Correlations between *empathy* and *familiarity with the music* and between *empathy* and perceived *beat clarity of the music* were nonsignificant (Figure 3(b) and (c)).

## Study 2

To increase the generalizability of the video paradigm, we extended the acoustic stimuli from one instrumental music clip and one metronome in Study 1 to three instrumental music clips and three metronomes in Study 2.

### Method

#### Participants

We analyzed data of 162 participants (mean age = 30.4 years, *SD* = 9.7; 114 female, 48 male). Fifty-eight participants were Danish; the other 104 participants had 41 different nationalities with the largest sub-samples being from Great Britain (*n* = 19), the United States (*n* = 8), and Germany (*n* = 6). Ten additional participants were excluded because they reported that they participated in a similar experiment before. Informed consent was provided and the study was approved by the IRB at the Danish Neuroscience Centre.

#### Stimuli and design

**Figure 4.**
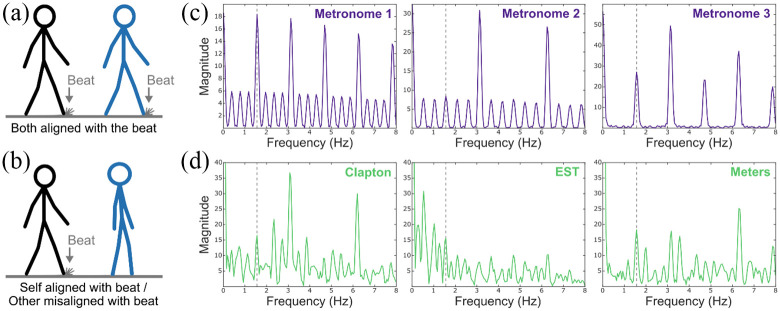
(a and b) The Two Different Movement Alignment Levels of the Social Entrainment Video Paradigm in Study 2. (c and d) Frequency Spectra of the Note-Onset Interval Series of the Three Metronome Stimuli (c) and the Three Music Stimuli (d) Detected by *mironsets* in the MIR Toolbox (Lartillot & Toiviainen, 2007) for MATLAB (MathWorks, Natick, MA). The Dashed Gray Line Represents the Beat of the Audio Stimuli and the Temporally Aligned Step Frequency of the Visual Stimuli, Which Was 94 bpm/638 ms.

Participants rated 12 videos with a length of 11 s each (see Supplemental material online). Similar to Study 1, these videos showed the following two walking stick figures: a black one representing the virtual self and a blue one representing the virtual other. The manipulated independent variables were *movement* and *music*. In contrast to Study 1, the variable *movement* had only the following two levels: (1) both figures aligned with the beat and (2) self aligned with the beat and other misaligned. The variable *music* had the following two levels: (1) music and (2) metronome (Figure 4; Supplemental material online). The musical pieces were instrumental excerpts of “My Father’s Eyes” by Eric Clapton (4/4 meter), “Elevation of Love” by Esbjörn Svensson Trio (6/8 meter; see Study 1), and “Thinking” by the Meters (4/4 meter). All musical pieces had a tempo of 94 bpm/638 ms (Figure 4(d)). The three metronomes consisted of 4/4, 6/8, and 2/4 meter at 94 bpm (Figure 4(c)). Identical to Study 1, temporally aligned figures had a step frequency of 94 bpm and the foot hit the ground on the beat of the music or the metronome. The moment of the foot hitting the ground was additionally marked by a “dust cloud” on the ground (see Figure 4). In contrast to Study 1, temporally misaligned figures started to walk with a step frequency of 78 bpm/769 ms with a 233 ms delayed first step, sped up to 86 bpm/698 ms, and slowed down to the start step frequency again.

#### Procedure and ratings

Similar to Study 1, participants watched each of the 12 videos in randomized orders and imagined that the black figure represented themselves and that the blue figure represented an unknown person (Stupacher et al., 2020; Stupacher, Maes, et al., 2017). They rated *IOS* and *well-being* in each video on a 100-point visual analogue scale. After the video rating, participants rated how much they enjoyed the music. At the end of the survey, participants filled out the Brief Form of the Interpersonal Reactivity Index (B–IRI; Ingoglia et al., 2016). As in Study 1, the mean of the B–IRI items was used as empathy value. Data were collected online on soscisurvey.de (SoSci Survey GmbH, Munich, Germany).

#### Data analysis

We modeled the dependent variables *IOS* and *well-being* in linear mixed effects models using the lmer function from *R*’s (R Core Team, 2018) lme4 package (Bates et al., 2015). All models allowed for a random intercept per participant to control for participant level variability of *IOS* and *well-being* ratings and for random intercepts for the six stimuli (three metronomes and three music excerpts). The full models included the following fixed effects: *Movement* (both aligned with the beat vs. self aligned/other misaligned), *music* (music vs. metronome), and *empathy*.

### Results

**Figure 5.**
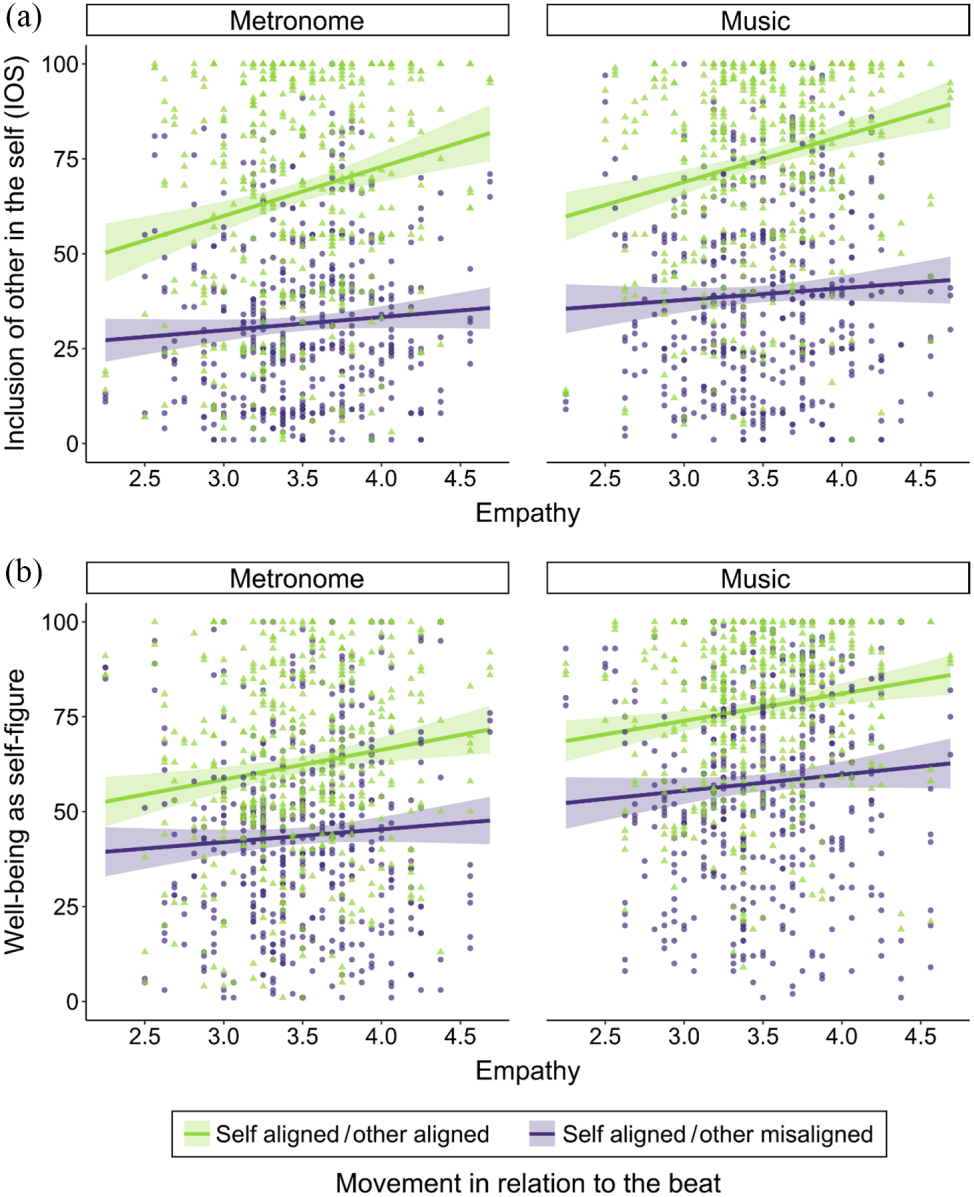
Individual Data Points and Linear Predictions of Interpersonal Closeness as Measured by *IOS* (Aron et al., 1992) and Well-Being in Relation to Participants’ Trait Empathy in Study 2. Shaded Areas Represent 95% Confidence Intervals. (a) *IOS* for the Two *Movement* Levels With Metronome and Music. (b) *Well-Being* for the Two *Movement* Levels With Metronome and Music.

For both dependent variables, *IOS* and *well-being*, ratings were higher when moving with a temporally aligned virtual other compared to a misaligned virtual other and higher for virtual movement interactions that featured music compared to metronomes. Participants with higher *empathy* rated *IOS* and *well-being* higher compared to participants with lower empathy. For IOS ratings, the best fitting model additionally indicated an interaction between *movement* and *empathy* (Figure 5(a) and Table 3). For *well-being* ratings, the best fitting model only included main effects of *movement*, *music*, and *empathy*; the interaction between *movement* and *empathy* tended to improve the fit of the model but was not significant, *p* = .063; Figure 5(b) and Table 3.

**Table 3.** Mixed Effects Models for the Dependent Variables *Inclusion of Other in the Self* (IOS) and *Well-Being* Investigating the Effects of the Independent Variables *Movement*, *Music*, and *Empathy* in Study 2.

Model	AIC	BIC	Marg. *R*^2^	Cond. *R*^2^	Model fit improvement	
					χ^2^(*df*)	*p*
Dependent variable: IOS						
M0: IOS null model	18,574	18,596		.248		
M1: IOS ~ movement (vs. M0)	17,449	17,477	.323	.601	1127(1)	<.001
M2: IOS ~ movement + music (vs. M1)	17,441	17,474	.341	.600	10.21(1)	.001
M3: IOS ~ movement × music (vs. M2)	17,443	17,482	.341	.600	0.17(1)	.681
M4: IOS ~ movement + music + empathy (vs. M2)	17,435	17,474	.354	.601	7.41(1)	.006
M5: IOS ~ movement + music × empathy (vs. M4)	17,437	17,482	.354	.601	0.09(1)	.762
M6: IOS ~ movement × music + empathy (vs. M4)	17,437	17,482	.354	.601	0.17(1)	.681
**M7: IOS ~ movement × empathy** **+** **music (vs. M4)**	**17,416**	**17,461**	**.358**	**.605**	**21.45(1)**	**<.001**
M8: IOS ~ movement × empathy × music (vs. M7)	17,422	17,483	.358	.605	0.28(3)	.965
Dependent variable: well-being						
M0: Well-being null model	17,789	17,811		.352		
M1: Well-being ~ movement (vs. M0)	17,358	17,386	.128	.492	432(1)	<.001
M2: Well-being ~ movement + music (vs. M1)	17,350	17,383	.203	.486	10.54(1)	.001
M3: Well-being ~ movement × music (vs. M2)	17,351	17,390	.203	.486	0.45(1)	.502
**M4: Well-being ~ movement** **+** **music** **+** **empathy (vs. M2)**	**17,347**	**17,386**	**.212**	**.487**	**4.69(1)**	**.030**
M5: Well-being ~ movement + music × empathy (vs. M4)	17,349	17,394	.212	.487	0.01(1)	.954
M6: Well-being ~ movement × music + empathy (vs. M4)	17,349	17,393	.212	.487	0.45(1)	.502
M7: Well-being ~ movement × empathy + music (vs. M4)	17,346	17390	.212	.488	3.45(1)	.063
M8: Well-being ~ movement × empathy × music (vs. M4)	17,351	17,412	.212	.488	4.06(4)	.398

AIC: Akaike information criterion; BIC: Bayesian information criterion; IOS: inclusion of other in the self.

All models included random effects for participants and stimuli. The Akaike information criterion (AIC), Bayesian information criterion (BIC), variance explained by fixed effects (marginal *R*^2^), and variance explained by fixed and random effects (conditional *R*^2^) are provided. χ^2^ and *p* values refer to model comparisons against models indicated in parentheses using likelihood ratio tests. The models that describe the data best are marked in bold.

The best fitting *IOS* model (*IOS* ~ *movement* × *empathy* + *music*) resulted in significant pairwise comparisons of *movement* (Misaligned—Aligned contrast estimate −35.2, *SE* = 0.88, *p* < .001, CI [−36.9, −33.4]), *music* (Metronome—Music contrast estimate −8.18, *SE* = 1.75, *p* = .010, CI [−13.1, −3.3]), and *empathy* (slope estimate 7.91, *SE* = 2.89, *p* = .007, CI [2.2, 13.6]). The interaction between *empathy* and *movement* was significant (Empathy|Misaligned—Empathy|Aligned estimate −9.24, *SE* = 1.99, *p* < .001, CI [−13.1, −5.3]).

The best fitting *well-being* model (*Well-being* ~ *movement* + *empathy* + *music*) resulted in significant pairwise comparisons of *movement* (Misaligned—Aligned contrast estimate −19.3, *SE* = 0.87, *p* < .001, CI [−21.0, −17.6]), *music* (Metronome—Music contrast estimate −14.60, *SE* = 3.19, *p* = .010, CI [−23.4, −5.7]), and *empathy* (slope estimate 5.65, *SE* = 2.60, *p* = .031, CI [0.5, 10.8]).

Similar to Study 1, *empathy* was positively correlated with the mean of the enjoyment of music, *r*(160) = .16, *p* = .049, indicating that higher empathy was associated with more enjoyment of the music.

## General discussion

Music is about humans interacting with each other; it is about communicating and understanding each other’s mental states and about coordinating and synchronizing each other’s movements and behaviors. These social interactions are key aspects of how music becomes meaningful. Our findings suggest that individual traits, such as empathy, can affect how we bond with others and how we feel in social interactions that feature music.

Overall, our results demonstrate that temporally aligned movements between virtual self and other increase social bonding and well-being. This positive effect of synchronized movement was found when virtual self and other were interacting with music or a metronome, supporting previous studies that showed a prosocial effect of interpersonal movement synchronization (Demos et al., 2012; Hove & Risen, 2009; Kokal et al., 2011; Stupacher et al., 2020; Stupacher, Maes, et al., 2017; Valdesolo & DeSteno, 2011; Wiltermuth & Heath, 2009). The consensus between these studies underlines the notion that prosocial consequences and positive emotional effects of moving to music in social settings are cultural universals and might be evolutionary adaptations (Savage et al., 2021; Trainor, 2015).

Our findings suggest that music plays a special role when moving in social settings. In both studies, videos in which virtual self and other were moving with music received higher ratings of social closeness compared to videos with metronomes. Additionally, both studies showed that when interacting with music, higher trait empathy was associated with stronger social bonding between virtual self and virtual other. However, two different interactions of social closeness ratings—an *empathy* × *music* interaction in Study 1 and an *empathy* × *movement* interaction in Study 2—revealed that the role of empathy in social interactions with music is multifaceted and may be influenced by subtle changes in the musical and social environment.

The *empathy* × *music* interaction of social closeness and well-being ratings in Study 1 suggests that individuals with high trait empathy may feel better and bond more with others when interacting with music compared to a simple timekeeper, such as a metronome. In contrast, individuals with lower trait empathy may feel better and bond more with others when interacting with a simple nonmusical timekeeper, which is connected to less social expectations and norms.

The *empathy* × *movement* interaction of social closeness ratings in Study 2 suggests that higher trait empathy may be associated with *strong* increases of social bonding when interacting with a temporally aligned other person, but only *weak* increases of social bonding with a temporally misaligned other person. In other words, when it comes to social bonding, individuals with less empathy may differentiate less between temporally aligned and misaligned others in interpersonal movement interactions compared to individuals with high empathy. A similar tendency can be seen in the ratings of well-being.

An explanation for why the *empathy* × *music* interaction of social closeness and well-being ratings was significant in Study 1 but not significant in Study 2 could be the selection of musical excerpts. Study 1 only featured an excerpt of the title “Elevation of Love” by Esbjörn Svensson Trio, which can be described as rhythmically driving but melancholic. Study 2, however, included a broader range of instrumental musical excerpts that, besides “Elevation of Love,” featured “Thinking” a groovy piece by The Meters, and “My Father’s Eyes,” a standard pop/rock piece by Eric Clapton. The *empathy* × *music* interaction in Study 1 might therefore represent the influence of empathy on social bonding with melancholic music. For music in general and for more uplifting music, this influence might be attenuated or even disappear. Huron and Vuoskoski (2020) argue that individuals who enjoy sad music more than others might have a specific pattern of trait empathy, which makes them experience pleasure through compassion and empathetic engagement. The results of Study 1 show that higher trait empathy was associated with more enjoyment of the excerpt from “Elevation of Love.” It is therefore possible that participants on the upper end of the trait empathy scale experienced more compassion with the music compared to the metronome, which could be reflected in stronger feelings of social closeness.

A potential reason for why we only found an interaction of social closeness ratings between *empathy* and *movement* in Study 2 is that Study 1 included three movement alignment levels, whereas Study 2 only included two movement alignment levels, which could have made it easier for participants to differentiate between temporally aligned and temporally misaligned movements. Additionally, the misaligned virtual other in Study 2 was not just moving at a different frequency and delayed, but was also speeding up and slowing down, which made the timing of the movements even less predictable and the misalignment even more obvious.

Future studies could directly manipulate the emotional responses that the music evokes to investigate the influence of specific patterns of trait empathy, such as compassion in individuals who particularly enjoy sad music, on social bonding in movement interactions. To better understand the influence of the clarity of movement alignment or misalignment, a finer manipulation of phase and frequency differences between self and other is needed; for example, aligned versus almost aligned versus slightly misaligned versus misaligned movements. Another open question is whether phase misalignment, frequency misalignment, or a combination of both most strongly affects ratings of social closeness.

Empathy does not only influence how we interpret social interactions with music, but joint musical activities can also promote empathy (Clarke et al., 2015; Greenberg et al., 2015), especially in individuals with empathy deficits (Behrends et al., 2012). Following the *empathy* × *music* interaction in Study 1, it may be beneficial to use isochronous or very simple rhythms in movement- and music-supported educational and clinical approaches to increase empathy. This conclusion is speculative, however, and not directly supported by Study 2, in which we did not find an interaction between *empathy* and *music*. The discrepancy between the two studies could mean that different types of music have different effects on social bonding in individuals with high or low empathy. Individuals with empathy deficits might therefore benefit most from selecting their own preferred music when joining group interactions that feature musical rhythm (Rabinowitch, 2015).

Empathy deficits are characteristic in individuals with autism spectrum disorder (ASD). In a study with a beat-based communication task, cognitive empathy toward a synchronous compared to asynchronous partner increased in neurotypical participants but not in participants with ASD (Koehne et al., 2016). The *empathy* × *movement* interaction in Study 2 is comparable to Koehne and colleagues’ findings; Participants with low empathy rated the social closeness toward temporally aligned and misaligned virtual others quite similar, whereas participants with high empathy rated social closeness toward the temporally aligned virtual other substantially higher than the misaligned virtual other.

A future opportunity is to explore physiological and psychological benefits of social musical activities involving synchronized movements for individuals with low trait empathy. Given the many facets of empathy and the complexity of social interactions with music, the collection of more empirical evidence is crucial. Accumulated insights gained from empirical studies, such as the current ones, can be applied to educational programs and clinical interventions that use joint musical activities to strengthen empathy and social bonding.

In both studies, trait empathy was positively correlated with enjoyment of the musical pieces in the virtual social interactions. This finding is in line with recent studies suggesting that trait empathy is associated with activity in prefrontal and reward areas in the brain when listening to music (Wallmark et al., 2018) and with greater enjoyment of dancing (Bamford & Davidson, 2019). A future challenge is to disentangle the close relationships between enjoyment of music and dancing, empathy, and social bonding, which could be achieved by choosing individual musical pieces for participants in joint musical activities and controlling for the enjoyment of these pieces. Importantly, our findings show that the perceived beat clarity of the used musical piece did not correlate with empathy, which makes it unlikely that differences in rhythm perception or beat induction influenced ratings of interpersonal closeness and well-being with music.

In sum, our findings provide insights into how our enjoyment, interpretation, and use of music in social situations may be related to our empathy traits. Both studies suggest that when moving together with music, higher empathy is associated with increased social bonding and well-being. Interactions of social closeness between empathy and the type of musical stimulus in Study 1 and movement alignment in Study 2 reveal that the interplay between these factors is not easily generalizable and may be influenced by small changes in the environment. We need to accumulate more evidence to fully understand how empathy influences the fascinating prosocial effects of moving together with music.
